# Successful treatment of acute hemoptysis of bronchiectasis by thoracoscopic bronchial artery ligation

**DOI:** 10.1186/1749-8090-8-S1-P27

**Published:** 2013-09-11

**Authors:** W Hsing-Hsien

**Affiliations:** 1 Department of Thoracic Surgery, Tainan Municipal Hospital, Tainan City, Taiwan

## Background

Acute massive hemoptysis is a life-threatening condition. Traditionally, bronchial artery embolization and lung resection are the treatment options. But there is high recurrence rate after bronchial artery embolization and some patients are not suitable for or can’t tolerate lung resection. We develop thoracoscopic bronchial artery ligation as another treatment option. This is the first report to apply this procedure in treatment of acute life-threatening hemoptysis in bronchiectasis.

## Methods

A 46-year-old male patient visited the emergency department due to cough up of bloody contents on December 14th in 2010. The hemoptysis ever developed 3 months ago and relieved after medical treatment. The reformatted imaging revealed engorged bronchial artery from descending aorta. Emergent thoracoscopic surgery was arranged. Bronchoscopy was done firstly after general anesthesia. Then bronchial artery ligation with clipping and wedge resection were performed. Bronchoscopy was performed again to clean out the blood clots.

## Results

The operative course was smooth. The postoperative recovery was uneventful. The chest was removed on the next day after surgery. The patient was discharged on the postoperative third day. The patient was followed till now. The hemoptysis no more attacked again.

## Conclusions

Life-threatening hemoptysis is an emergent condition. Traditionally bronchial artery embolization individual, pulmonary resection or both are the treatment options. The procedure is safe and effective. This procedure individual or combined with pulmonary resection may become a new option in treatment of acute hemoptysis.

